# Forecasting sub-national trends in COVID-19 vaccine uptake in the UK before vaccine rollout

**DOI:** 10.1038/s41598-022-25354-4

**Published:** 2022-12-13

**Authors:** A. de Figueiredo

**Affiliations:** 1 Department of Infectious Disease Epidemiology, London School of Hygiene and Tropical Medicine, London, UK; 2 Department of Mathematics, Imperial College London, London, UK

**Keywords:** Infectious diseases, Health policy, Public health

## Abstract

Vaccines have reduced the burden of COVID-19 disease in the UK since their introduction in December 2020. At the time of their introduction, it was unclear the extent to which COVID-19 vaccines would be accepted and how spatial variations in uptake would emerge, driven by socio-demographic characteristics. In this study, data from a large-scale cross-sectional study of over 17,000 adults, surveyed in September and October 2020, was used to provide sub-national forecasts of COVID-19 vaccine uptake across the UK. Bayesian multilevel regression and poststratification was deployed to forecast COVID-19 vaccine acceptance before vaccine rollout across 174 regions of the UK. Although it was found that a majority of the UK adult population would likely take the vaccine, there were substantial heterogeneities in uptake intent across the UK. Large urban areas, including London and North West England, females, Black or Black British ethnicities, and Polish speakers were among the least likely to state an intent to vaccinate. These predicted spatial trends were validated by comparison to observed observed COVID-19 vaccine uptake in late 2021. The methodological approaches deployed in this validated forecasting study may be replicable for the prediction of routine childhood immunisation uptake. Given recent pandemic-induced disruptions to routine immunisation systems, reliable sub-national forecasts of vaccine uptake may provide policymakers and stakeholders early warning signals of potential vaccine confidence issues.

## Introduction

A vaccine against COVID-19 disease caused by the severe acute respiratory coronavirus 2 (SARS-CoV-2) has been a major step in reducing associated global mortality, morbidity, economic, and societal burdens. In the United Kingdom (UK), the first COVID-19 vaccine was administered on 9 December 2020 and by January 2021–a year after the first recorded COVID-19 case in the UK^1^– the UK’s National Health Service (NHS) had begun the rollout of two vaccines approved by the Medicines and Healthcare products Regulatory Authority (MHRA)^2^, with a further four approved as of June 2022^3^.

Successful COVID-19 vaccination campaigns have relied on several factors: at-scale manufacturing ensuring sufficient dosages; fast and equitable vaccine distribution and supply via existing and novel supply-chain networks with sufficient capacity for storage and delivery; and public confidence in the vaccine and the systems advising and delivering them. This latter factor was (and still is) of particular concern in the UK. The UK has experienced notable hesitancy towards some immunisations^4^, including recent year-on-year decreases in the uptake of routine immunisations such as the MMR vaccine^5^ with corresponding outbreaks resulting in the loss of the UK’s measles-free status^6–9^. Destabilising vaccine confidence further is false information around vaccines, which has been prominent during the COVID-19 pandemic, including before COVID-19 vaccine rollout in the UK^10–12^.

In this modelling study, intent to accept a COVID-19 vaccine was estimated for 174 sub-national regions across the UK using a cross-sectional online survey. Data from over 17,000 adults were collected between 24 September and 14 October 2020, reduced to 16,820 after data quality control procedures (see Methods). Multilevel regression and poststratification (MRP))^13,14^ with an ordinal logistic multilevel model was used to identify the socio-demographic barriers of intent to accept a COVID-19 vaccine and obtain sub-national forecasts of vaccination intent before the COVID-19 vaccine was rolled out across the UK. Socio-demographic data was collected for each respondent to assess the relationship between these characteristics and vaccine intent and to allow for poststratification using UK census microdata records containing the same socio-demographic information^15^. Individuals’ sex, age, highest educational attainment, religious affiliation, ethnicity, employment status, primary language, and outer postcode were collected. Outer postcode–the first half of a UK postcode–was required to map respondents to one of 174 third level units of the classification of Eurostat's nomenclature of territorial units for statistics (NUTS-3). Descriptions for all respondent data collected and recodings are provided in Table 1. Model validation was performed by correlating MRP forecasts of vaccine uptake values at the regional level against observed first dose uptake rates reported by the NHS on 23 September 2021. The use of first-dose uptake data on 23 September allowed for at least three full months to elapse since all adults had been offered at least one COVID-19 vaccine^16^. (See Methods for a full description of data collection, data quality control processes, and MRP.) All analyses in this study, with the exception of model validation, was performed before vaccine rollout in the UK^17^.

**Table 1:** Study data Survey items are shown with possible responses (including recodes, if any), and baselines used in the ordinal multilevel logistic regressions. COVID-19 vaccination intent is the study response variable.

Survey question	Values (recodes, including to align with UK census in parenthesis)	Regression baseline
**Response: COVID-19 vaccination intent**		
If a new coronavirus (COVID-19) vaccine became available, would you accept the vaccine for yourself?	Yes, definitely; unsure, but leaning towards yes; unsure but leaning towards no no, definitely not	n/a (response variable)
**Covariates: socio-demographic factors**		
Sex	Male and female	male
Age	Integer value mapped to 18–24, 25–34, 35–44, 45–54, 55–64, 65–79, 80 +	18–24
Highest educational attainment	No academic qualifications (none/other) 0–4 GCSE, O-levels, or equivalents (level 1–3) 5 + GCSE, O-levels, 1 A level, or equivalents (level 1–3) 2 + A levels or equivalents (level 1–3) Undergraduate or postgraduate degree or other professional qualification (level 4) Apprenticeship (none/other) Other (e.g., vocational, foreign qualifications) (none/other) Do not know (none/other) Do not wish to answer (none/other)	Level 1–3
Religious affiliation	Atheist/agnostic Christian Buddhist (other religion) Hindu Muslim other religion do not wish to answer (not given)	Atheist/agnostic
Work status	Working full-time (including self-employed) part-time (including self-employed) unemployed student Looking after the home Retired (retired / disabled) Unable to work (e.g., short- or long-term disability) (retired / disabled) Do not wish to answer (other work status)	Full-time
Ethnicity	White: English/Welsh/Scottish/Northern Irish/British (White) White: Irish (White) White: Other white background (White) White and Black Caribbean (mixed) White and Black African (mixed) White and Asian or White and Asian British (mixed) Black, African, Caribbean or Black British (Black/Black British) Asian or Asian British: Indian (Asian/Asian British) Asian or Asian British: Pakistani (Asian/Asian British) Asian or Asian British: Chinese (Asian/Asian British) Asian or Asian British: Other (Asian/Asian British) other ethnicity (other ethnicity) do not wish to answer (other ethnicity)	White
Language	English or Welsh Polish Punjabi (other language) Urdu (other language) Bengali (other language) Other (other language) do not wish to answer (other language)	English or Welsh

Although studies preceding data collection in this study revealed largely positive attitudes towards a COVID-19 vaccine across the UK^18,19^, no study has forecasted local-level trends in COVID-19 vaccine acceptance. Although the acquisition of herd immunity against SARS-CoV-2 seems unlikely due to waning immunity^20,21^, this study provides a proof-of-principle for spatial forecasting of vaccine acceptance that could be used–for vaccines conferring sterilising immunity–to identify vaccine confidence “cold spots” that could disproportionately increase required vaccination levels to achieve herd immunity in ‘adjacent’ settings^22,23^.

## Results

Respondents were asked whether they would accept a COVID-19 vaccine: “*If a new coronavirus (COVID-19) vaccine became available, would you accept the vaccine for yourself?*”. Respondents could provide one of four responses on an ordinal scale: “*yes, definitely*”, “*unsure, but leaning towards yes*”, “*unsure, but leaning towards no*”, or “*no, definitely not*”.

Throughout this section, estimates of quantities are provided as a mean value with a 95% highest posterior density interval (unless otherwise stated). The highest posterior density interval (HPDI) is the shortest interval of the posterior distribution that contains 95% of the probability mass.

### MRP estimates of COVID-19 vaccination intent

**Figure 1:**
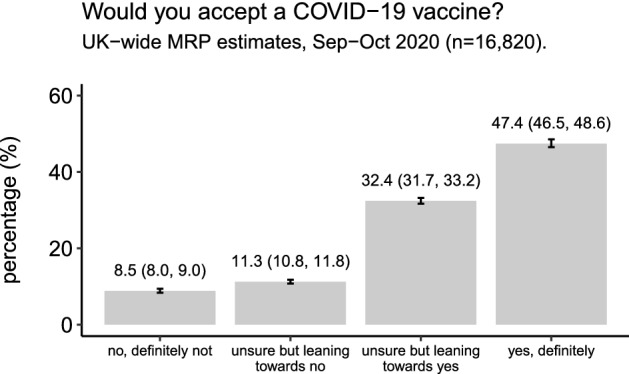
National-level estimates of COVID-19 vaccine uptake intent. National-level estimates for the percentage of the UK that would “definitely not” accept a COVID-19 vaccine, “definitely” accept a COVID-19 vaccine, or who are unsure. Mean MRP estimates with 95% highest posterior density intervals are provided.

Across the UK, just under half the population, 47.4% (95% HPDI 46.5–48.6%) reported they would “definitely” take a COVID-19 vaccine, while a further 32.4% (31.7–33.2%) were leaning towards vaccinating but were unsure. 8.5% (8.0–9.0%) reported they would “definitely not” take a COVID-19 vaccine and 11.3% (10.8–11.8%) were unsure but leaning towards no (Fig. 1).

**Figure 2:**
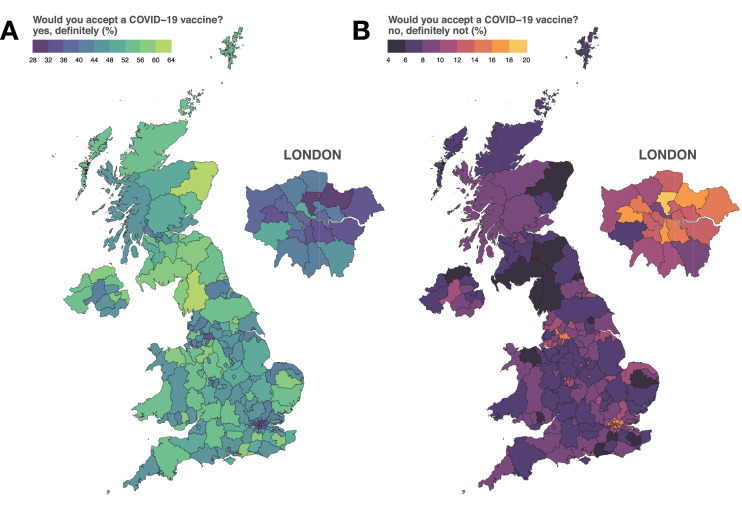
Sub-national estimates of COVID-19 vaccine intent across the UK. The estimated proportion of respondents in each of the UK’s 174 NUTS-3 region who state they would “definitely” accept a COVID-19 (**A**) and “definitely not” accept a COVID-19 vaccine (**B**). Regional boundaries are used under the Open Government License v3.0 (see https://data.gov.uk/dataset/b147a160-86b6-48e4-8dd0-f35b90981814/nuts-level-3-january-2015-super-generalised-clipped-boundaries-in-england-and-wales accessed 25 November 2020).

**Figure 3:**
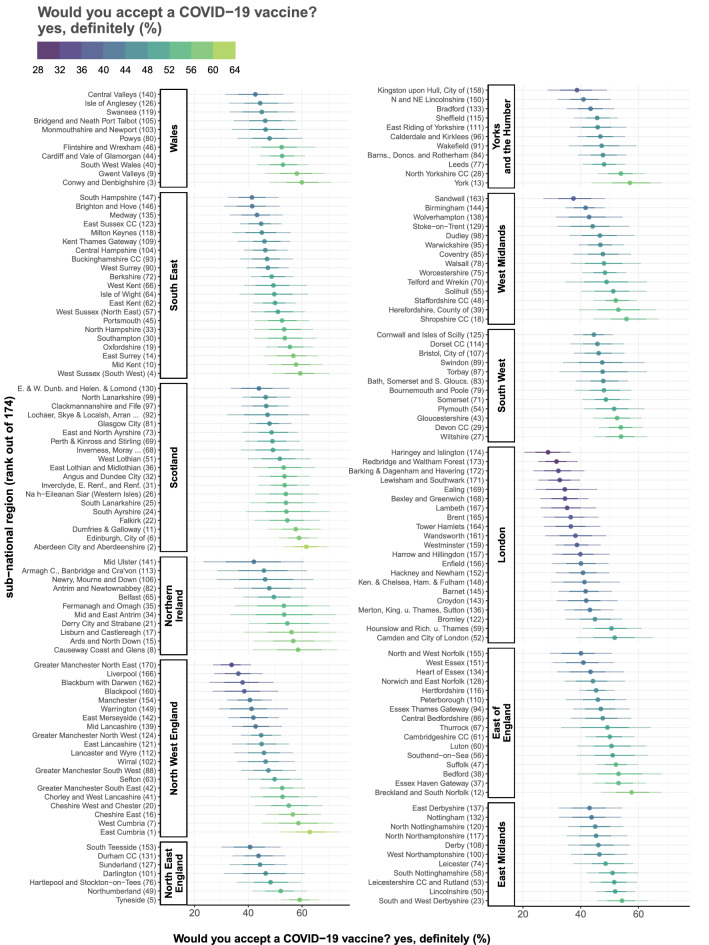
Intent to accept a COVID-19 vaccine ranked within each NUTS-1 region. The MRP estimated proportion of respondents in each of the UK’s 174 NUTS-3 region who would definitely accept a COVID-19 vaccine are shown and ranked within the 12 first-level NUTS regions (NUTS-1). 70% and 95% highest posterior density intervals (horizontal bars) are shown around the mean estimate (dot). Each region is suffixed by its rank (out of 174) according to the estimated proportion who would “definitely” accept a COVID-19 vaccine. East Cumbria (North West England) ranked first, while Haringey and Islington (London) ranked last.

Sub-national MRP estimates of the proportion of each of the UK’s 174 NUTS-3 regions who reported they would “definitely” accept a COVID-19 vaccine are mapped in Fig. 2A. Estimates of the proportions who reported they would “definitely not” accept a COVID-19 vaccine are mapped in Fig. 2B. The values in Fig. 2A are repeated in Fig. 3 with their corresponding 70% and 95% HPDIs and are ranked from lowest to highest acceptance by NUTS-1 region. (NUTS-1 is the first NUTS level.)

Estimates across the 174 sub-national NUTS regions of the UK varied considerably. Estimates of the proportion of the public who stated they would “definitely” accept a COVID-19 vaccine (Fig. 2A) ranged from 28.7% (20.5–36.6%) in Haringey and Islington to 62.9% (51.9–73.7%) in East Cumbria (Figs. 2, 3). The lowest proportions of the UK public stating they would “definitely” accept a COVID-19 vaccine were concentrated in London, which contains 13 of the 20 lowest proportions in the UK: Haringey and Islington 28.7% (20.5–36.6%); Redbridge and Waltham Forest (31.6%, 25.3–38.9%); Barking & Dagenham and Havering (32.3%, 24.6 to 41.3%); Lewisham and Southwark (32.7%, 25.3–39.7%); Ealing (34.5%, 24.4–45.6%); Bexley and Greenwich (34.5%, 26.0 to 42.6%); Lambeth (35.2%, 26.1 to 45.3%); Brent (36.5%, 27.1–46.1%); Tower Hamlets (36.6%, 27.3–46.8%); Wandsworth (38.2%, 27.8–48.7%); Westminster (38.7%, 31.2 to 46.9%); Harrow and Hillingdon (39.9%, 30.2–49.9%); and Enfield (40.0%, 30.2–49.5%). Four of the remaining seven regions in the lowest 20 were in North West England: Greater Manchester (33.8%, 26.9–41.0%); Liverpool (36.3%, 27.2–45.3%); Blackburn with Darwen (37.9%, 25.6 to 49.4%), and Blackpool (38.5%, 26.8–51.1%). The remaining three areas in the lowest 20 are North and West Norfolk (East of England, 40.1%, 29.4–50.8%), Sandwell (West Midlands, 37.5%, 27.1–48.4%), and the City of Kingston upon Hull (Yorkshire and the Humber, 38.7%, 28.6–49.1%). (See supplementary data file for all estimated values and posterior intervals.)

The five regions with the highest proportions of the UK public who would “definitely” accept a COVID-19 vaccine were East Cumbria (62.9%, 51.9–73.7%), Aberdeen City and Aberdeenshire (61.6%, 53.5 to 69.4%), Conwy and Denbighshire (59.9%, 48.0–70.7%), West Sussex (59.3%, 49.1–70.2%), and Tyneside (59.1%, 52.1–66.4%).

The regions with the highest estimated proportions who would “definitely not” accept a COVID-19 vaccine were again predominately located in London and the North West. Haringey and Islington (19.5% (12.8 to 26.5%), Redbridge and Waltham Forest (17.7, 12.4–23.2), and Lambeth (16.4%, 10.1–23.1%) have the highest estimated proportions who would “definitely not” accept the vaccine, while East Cumbria (4.2%, 2.3–6.4%), Aberdeen City and Aberdeenshire (4.4%, 2.8–6.1%), and Tyneside (4.9%, 3.4 to 6.4%) had the lowest. Estimates for the proportions of respondents who are “unsure” about taking a COVID-19 vaccine are mapped in the Supplementary Materials, Fig. S1.

### Socio-demographic determinants of vaccination intent

**Figure 4:**
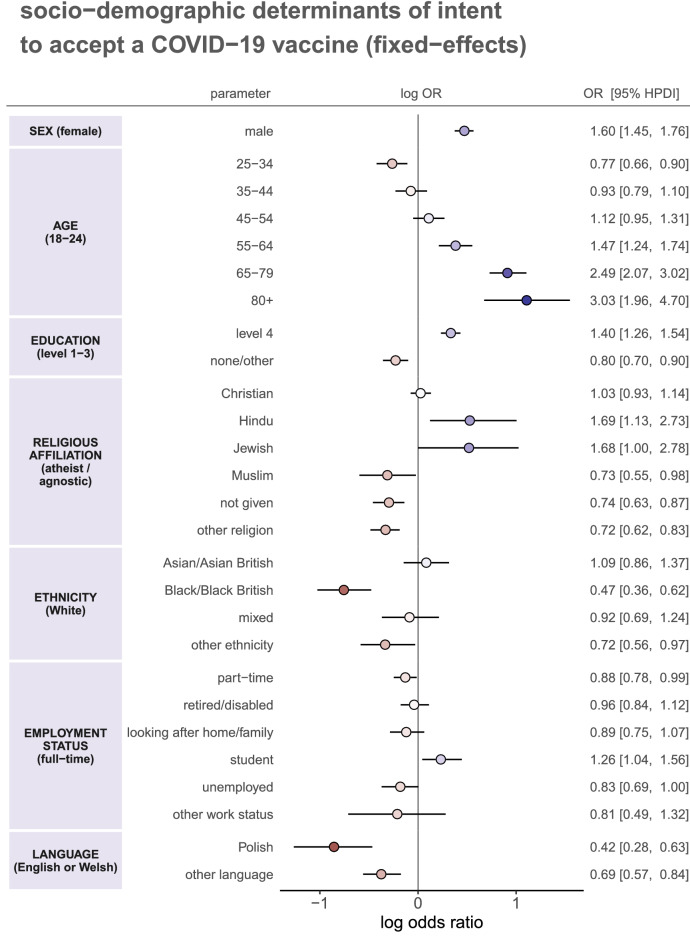
Socio-demographic determinants of intent to accept a COVID-19 vaccine. Multilevel regression fixed-effect parameter log odds ratios are plotted with corresponding 95% HPDIs. These log odds ratios are coloured by effect magnitude and direction, where blues (reds) signify that the group is more (less) likely than the baseline group to accept a COVID-19 vaccine. The darker the colour the stronger the association. For each factor, the baseline group is provided in parentheses on the left. Odds ratios with 95% HPDIs are shown on the right for each parameter.

The fixed-effects in the ordinal multilevel regression (see Methods)–which represent an “average” impact of socio-demographic characteristics on vaccination intent across the whole country–are shown in Fig. 4.

Several factors were found to be associated with COVID-19 vaccine intent. Males were more likely than females (odds ratio 1.60, 95% HPDI 1.45–1.76) to state intent to accept a COVID-19 vaccine. Older age groups were more likely to state acceptance than 18–24-year-olds, in particular 65–79 and 80 + year-olds (2.49, 2.07–3.02 and 3.03, 1.96–4.70, respectively). Interestingly, 25–34-year-olds were *less* likely than 18–24-year-olds to state intent to accept (0.77, 0.66–0.90). Individuals with undergraduate or postgraduate qualifications (level 4) were more likely than those with GCSEs, A- or O-levels to state intent to accept (1.40, 1.26–1.54) while those with no formal qualifications or other qualifications (see Table 1) were less likely (0.80, 0.70–0.90).

Those who identify as Christian were as likely as atheists or agnostics to state intent to accept a vaccine (1.03, 0.94–1.12), but those reporting Hinduism or Judaism as their religion were more likely than atheists or agnostics to be willing to accept a COVID-19 vaccine (1.69, 1.13–2.73 and 1.68, 1.00–2.78, respectively). Those identifying as Muslim (0.73, 0.55 to 0.98), those not providing their religion (0.72, 0.62–0.83), or stating another (“other”) religious affiliation (0.72, 0.62–0.83) were less likely to accept a COVID-19 than atheists or agnostics. Ethnicity also plays a role in determining intent to accept a COVID-19 vaccine, independently of religion, with those identifying as Black or Black British (0.47, 0.36–0.62) and those reporting another (“other”) ethnicity (0.72, 0.56–0.97) less likely to state intent to accept a COVID-19 vaccine than Whites.

Individuals’ employment status appears to have played less of a role than the other factors outlined above, with odds ratios closer to one. However, there is evidence to suggest that those in part-time work (0.88, 0.78–0.99) or unemployed individuals (0.83, 0.69–1.00) were less likely than those in full-time in employment to state intent to accept a COVID-19 vaccine, while students (1.26, 1.04–1.56) were more likely.

Individuals who reported Polish (0.42, 0.28–0.63) or another language besides English or Welsh (0.69, 0.57–0.84) were less likely to state an intent to accept a COVID-19 vaccine than those who reported English or Welsh as their primary language.

### Model validation against recent English uptake data

Vaccination rollout began in the UK on 8 December 2020 and, as of June 2021, all adults in the UK had been offered a COVID-19 vaccine. The MRP NUTS-3 vaccine estimates were correlated against observed vaccine uptake data derived by dividing reported first-doses administered by NHS England by National Immunisation Management System (NIMS) population estimates^24^. (Throughout COVID-19 vaccine rollout, the NHS provided weekly updates on the number of doses administered in each Local Tier Local Authority, which can be readily mapped to NUTS-3 region.) A Bayesian linear correlation coefficient was calculated between logit-transformed observed first-dose coverage reported by the NHS and the percentage of respondents stating they would “definitely” vaccinate or who are “unsure, but leaning towards yes” across all NUTS-3 regions. (A logit transform is used to transform data confined to the range [0, 100] to the real line and, in this case, is given by $-\mathrm{log}\left(\frac{100}{y}-1\right)$, where $y\in (0, 100)$.)

**Figure 5:**
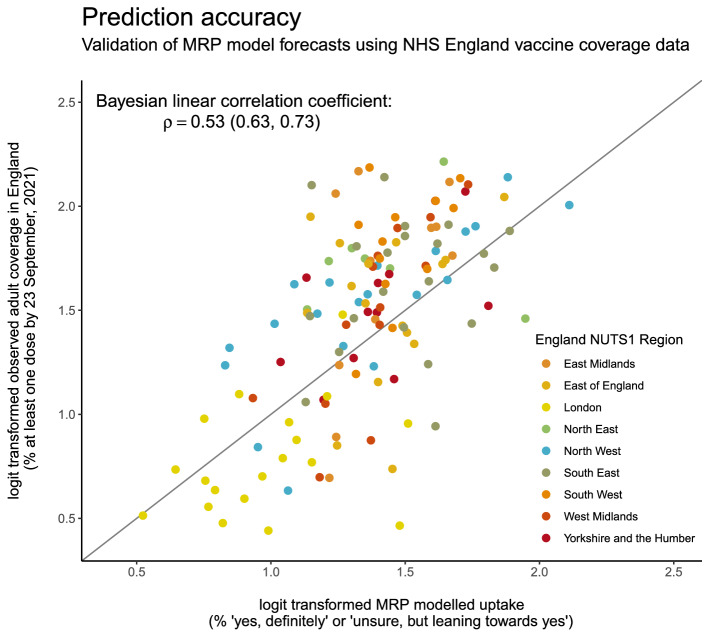
MRP forecasts of vaccine acceptance correlate with observed first-dose COVID-19 vaccine uptake among adults. (Logit transformed) observed vaccination coverage across all England NUTS-3 regions is plotted against (logit transformed) MRP predicted vaccine uptake. NUTS-3 regions are coloured by first administrative region (NUTS-1). The identity line is shown for reference.

Predicted vaccine acceptance (the percentage) across all NUTS-3 regions in England correlates with observed uptake reported by the NHS, with a Bayesian linear correlation of $\rho$=0.53 (0.63, 0.73), see Fig. 5.

## Discussion

Confidence in the COVID-19 vaccine has been extensively studied over the past two years^25–30^. In global studies of COVID-19 vaccine acceptance, the UK has had high reported confidence compared to other European countries^25,26,29^, which has translated to observed vaccine uptake data^31^. This study differed from previous studies by seeking to forecast sub-national trends in COVID-19 vaccine acceptance in advance of vaccine rollout.

Overall, a total of 47.4% (46.5–48.6%) of adults stated that they would ‘definitely’ receive a COVID-19 vaccine, yet on 2 June 2021, three quarters of adults had already received at least one COVID-19 vaccine^32^, suggesting improved population-level confidence in the COVID-19 vaccine in the UK. As of June 2022, 93% of people aged over 12 have received at least one dose of a COVID-19 vaccine^33^. A study of over 30,000 adults in the UK conducted between 7 September to 5 October 2020 found similar rates of intent to reject a vaccine, though direct comparison is difficult due to differences in questionnaire wording^19^.

This study found strong regional variation in intent to accept a COVID-19 vaccine in advance of vaccine rollout in the UK in December 2020. Although a relatively small proportion of the UK adult population (8.5%, 8.0–9.0%) stated that they would “definitely not” accept a vaccine, rates of intent to reject a COVID-19 vaccine were much higher in London and the North West, where they reached as high as 19.5% (12.8–26.5%) in Haringey and Islington. This strong regional variation predicted by the MRP modelling approach in this study has been borne out by observed vaccine uptake data, in which we find a strong correlation across all English regions between observed and predicted uptake. As of September 2021, three months after all adults had been offered a COVID-19 vaccine, London had 16 of the 20 English NUTS-3 regions with the lowest first dose uptake. Predicted coverage was in fact *higher* than observed uptake in all but one of these regions, whereas outside this lowest 20, predicted uptake was *lower* than observed uptake in three quarters of English regions. This result is strongly suggestive of persistently low COVID-19 vaccine confidence in London.

The contrast between London and national trends could be due to the interaction of between socio-demographic groups and UK vaccination policy. This study found that socio-demographic background is strongly associated with intent to accept the vaccine, with females, younger age groups, Muslims and ‘other’ religions, and Black and ‘other’ ethnicities, and non-English speakers less likely to state an intent to vaccinate than their respective baseline groups. While many of these associations had been found at the time of this analysis for both existing immunisation programmes^34–37^ and a COVID-19 vaccine^19,38,39^, recent evidence has suggested that COVID-19 vaccine passports may drive lower vaccination sentiment in the UK^40,41^. In particular, lower sentiment is evidenced in groups with low COVID-19 vaccine confidence, which includes young adults, males, those with Black/Black British ethnicity (a result also found in US populations^42^), as well as highly educated groups^40^: this is a plausible explanation for London’s MRP forecasts lagging observed uptake, opposing broader national trends. Vaccine passport policies have typically been found to be polarising, increasing uptake intentions in some groups but reducing them in others^40,42^: of particular concern in ongoing and future vaccination campaigns is whether these one-size-fits-all policies have enhanced resistance in areas with low vaccine confidence, which may pose challenges for achieving local and national herd immunity targets^22^.

As of 10 June 2022, third-dose vaccination rates were lower among younger age groups and Black ethnicities, aligning with findings from this study^43^. However, in the period 8 December to 11 March 2021 females aged over 70 were found to have higher coverage than their male counterparts^44^, a reversal of the association between sex and vaccine acceptance found in this study.

### Limitations

There are several study limitations to note. A key limitation of this present study is that the precise drivers of COVID-19 vaccine acceptance beyond socio-demographic determinants have not been considered. In the recent published literature in the UK and beyond, there are a wide-range of possible reasons for non-vaccination that includes trust in the COVID-19 vaccines themselves or in those recommending the vaccine^27^, the impact of misinformation^10^, or the impact COVID-19 vaccination policies on intent-to-vaccinate behaviours^40,41^. Extending the set of putative determinants may have yielded more robust forecasts of COVID-19 vaccine acceptance. Further, census data used in this study are a decade old^15^. Demographic changes in the UK between 2011 and 2020 within the regional populations studied could, therefore, result in biased estimates of vaccine intent. This study was also conducted via an online survey panel; while efforts were made to ensure representativeness via MRP, there may be biases arising from technological literacy or access to technology.

Despite these study limitations, this study provides robust forecasts of uptake of a novel immunisation programme at regional scales consistent with those relevant for local policymaking or for improving epidemiological projections of COVID-19 mortality in the UK^45^. Given recent global declines in routine immunisation rates^46^ and corresponding re-emergence of vaccine-preventable diseases^47^, this study demonstrates that future predictions of immunisation acceptance rates at epidemiologically and policy-relevant spatial resolutions is possible and can provide early warning signals of local vaccine confidence cold-spots.

## Methods

### Data collection

Between 24 September and 14 October 2020, a cross-sectional online survey (see Supplementary Materials) probing acceptance of a novel COVID-19 vaccine was administered to 17,684 UK residents aged 18 and over. Informed consent was obtained from all respondents before the survey commenced. During data collection, quality control procedures resulted in the removal of 864 respondents.

The initial sample size was chosen to maximise the number of observations within each of the sub-national regions: this study has approximately 100 observations for each of the 174 sub-national regions, which far exceeds sample sizes used in similar research^48^. Respondent quotas were set according to national demographic distributions for sex, age, and sub-national region (the second level of the Nomenclature of Territorial Units for Statistics, or ‘NUTS2’, see https://www.ons.gov.uk/methodology/geography/ukgeographies/eurostat accessed 25 November 2020) and which were re-adjusted based on the removal of respondents through the ongoing quality control checks during data collection. These quotas ensured a geographic spread of respondents across the UK, between the sexes, and across all age groups. All respondents were recruited via an online panel by ORB International (www.orb-international.com) and informed consent was obtained before respondents participated.

The response variable is whether a respondent would accept a COVID-19 vaccine: “*If a new coronavirus (COVID-19) vaccine became available, would you accept the vaccine for yourself?*”, with responses on a four-point ordinal scale: “*yes, definitely*”, “*unsure, but leaning towards yes*”, “*unsure, but leaning towards no*”, or “*no, definitely not*”. The rationale behind this choice of responses is to elicit an explicit vaccination intent rather than provide a continuous or Likert scale, from which the intent to vaccinate may be less clear.

Covariate data are the socio-demographic traits collected for each individual. These covariate data were chosen to align with the latest UK census: sex, age, highest educational attainment, religious affiliation, ethnicity, employment status, primary language, and outer postcode. Respondent’s outer postcode was used to map respondents to one of 174 third level NUTS regions (NUTS-3). The maximum number of surveys conducted in a NUTS-3 region is 293 (Hertfordshire) and the minimum is 16 (Mid and East Antrim). The mean number of responses per NUTS-3 unit is 96.7 (with standard deviation 52.1) and the median is 85. A breakdown of the number of individuals surveyed by socio-demographic characteristic is found in Supplementary Materials, Fig. S1 and the survey counts for each NUTS-3 region can be found in the supplementary data file.

### Multilevel regression and poststratification

Multilevel regression and poststratification (MRP) was used to estimate opinions aggregated at sub-national regions from survey data collected at the national level, via partial pooling of information between these national and sub-national scales^49^. This pooling of information between the two levels is a compromise between estimates derived via a total aggregation of data (to estimate national trends only) and estimates via complete disaggregation (that is, estimating regional trends only). The former suffers from a loss of information at the regional level while the latter suffers from possible low data counts and the loss of statistical power. More pooling of information will occur in regions with low relative numbers of surveyed individuals and less pooling in regions with high relative counts.

In brief (and relating specifically to this study), the first step of MRP is to conduct a multilevel regression to estimate, for each stratum (that is, a possible combination of individual characteristics) and for each region, the probability of COVID-19 vaccine acceptance. The second step is to reweight (post-stratify) these strata probabilities by the frequency with which a given strata appears in a population. In this study individual-level UK census data is used to perform the reweighting.

### Part 1: Multilevel regression

Individual intent to accept a COVID-19 vaccine is specified as ${y}_{ij}\in \{\mathrm{1,2},\mathrm{3,4}\}$, where 1 = “no, definitely not”, 2 = “unsure, but leaning towards no”, 3 = “unsure, but leaning towards yes”, and 4 = “yes, definitely” so that ordering is imposed on the response variables. Here, $j=1, \dots , 174$ is one of the $J=174$ third National Territorial Units for Statistics (NUTS-3) regions in the UK, and $i=1, \dots , {n}_{j}$, where ${n}_{j}$ is the number of individuals surveyed in region $j$. ${\sum }_{j}{n}_{j}=\mathrm{16,820}$ is the total number of respondents in the survey. A breakdown of the number of respondents in each region and a summary of their socio-demographic status is given in the supplementary data file.

Intent to accept a COVID-19 vaccine is modelled as a multilevel ordinal regression with the proportional odds assumption^50^,

$$Y_{ij} |{\varvec{p}}_{ij} , n_{j} , \sim {\text{Multi}}\left( {{\varvec{p}}_{ij} , 1} \right)$$

$$\begin{aligned} \log \frac{{\Pr \left( {Y_{ij} \le k|x_{ij} } \right)}}{{\Pr \left( {Y_{ij} > k|x_{ij} } \right)}} = & \rho_{k} + \beta_{j \, SEX\left[ i \right]} + { }\beta_{j \, AGE\left[ i \right]} { } + { }\beta_{j \, EDU\left[ i \right]} { } + { }\beta_{j \, REL\left[ i \right]} { } + { }\beta_{j \, ETH\left[ i \right]} + { }\beta_{j \, EMP\left[ i \right]} + { }\beta_{j \, LAN\left[ i \right]} { } \\ {\text{for }}k = & 1, \ldots ,3, \\ \end{aligned}$$

where ${\beta }_{j\, SEX\left[i\right]}$, ${\beta }_{j \, AGE\left[i\right]}$, ${\beta }_{j \, EDU\left[i\right]}$, ${\beta }_{j \, REL\left[i\right]}$, ${\beta }_{j \, ETH\left[i\right]}$, ${\beta }_{j \, EMP\left[i\right]}$, and ${\beta }_{j \, LAN\left[i\right]}$ are the random-effect varying intercepts for sex, age, highest education level, religious affiliation, ethnicity, employment status, and primary language (respectively);${\rho }_{k}$ are probability threshold parameters; $k\in \{\mathrm{1,2},\mathrm{3,4}\}$ is the ordinal response category; ${{\varvec{p}}}_{ij}=[\mathrm{Pr}\left({Y}_{ij}=1\right),\mathrm{Pr}\left({Y}_{ij}=2\right),\mathrm{Pr}\left({Y}_{ij}=3\right),\mathrm{Pr}\left({Y}_{ij}=4\right)]$; and ${x}_{ij}$ is a (dummy-coded) vector of  covariate data for individual $i$ in region $j$, that is reflected through the parameter superscripts. The baseline group for the regression corresponds to an individual who is male, aged 18–24, has an education level 1–3, is an atheist or agnostic, is White, works full-time, and speaks English or Welsh as their primary language (see Table 1). The random-effect parameter for each baseline category is zero and is accounted for in the probability threshold parameters.

In line with prior recommendations for variance components in hierarchical models^49,51^, default weakly informative priors are chosen for the random-effects regression coefficients $\beta$, (Instead of an noninformative ${\mathrm{N}}_{+}\left(0,100\right)$ distribution over the standard deviation of hierarchical variance parameters^51^, a weakly-informative ${\mathrm{N}}_{+}\left(0,1\right)$ prior is placed over the *precision* of these parameters, which places 95% of ${\sigma_l}$’s prior mass between 0.54 and 4.05.)

$$\begin{aligned} \beta_{lj} |\sigma_l \sim & {\text{N}}\left( {\gamma_l ,{ }\sigma_l^{2} } \right), \\{\sigma_l }^{-2} = & \tau_l \sim {\text{N}}_{ + } \left( {0,1} \right), \\ \beta_{lj} \sim & {\text{N}}\left( {0,1} \right),\;\;\;\;\;\;\;\;\;\;\;\, \\ \end{aligned}$$

where $l$ indexes the 27 non-baseline regression coefficients: $SEX = \{\textrm{female}\}$, $AGE = \{25-34, 35-44, 45-54, 55-64, 65-79,80+\}$, $EDU=\{\textrm{level 4}, \textrm{none \ / other}\}$, $REL = \{\textrm{Christian}, \textrm{Hindu}, \textrm{Jewish}, \textrm{Muslim}, \textrm{Not \ given}, \textrm{Other} \}$, $ETH = \{\textrm{Asian \ / Asian \ British}, \textrm{Black \ / Black \ British}, \textrm{Mixed \ ethnicity}, \textrm{Other} \}$, $EMP = \{\textrm{Part \ time}, \textrm{Retired/Disabled}, \textrm{Student}, \textrm{Looking \ after \ the \ home}, \textrm{Unemployed}, \textrm{Other}\}$, and $LAN = \{\textrm{Polish}, \textrm{Other}\}$.

### Part 2: Post-stratification

There are $S=$ 30,870 socio-demographic strata (two sexes × seven age groups × three education levels × seven affiliations for religion × five ethnicity groupings × seven employment statuses × three languages). Denoting the posterior distributions of COVID-19 vaccination intent for each stratum $s=1, \dots , S$ and NUTS-3 region $j=1, \dots , J$ as ${\theta }_{sjk}$ (where, as a reminder, $k\in \{\mathrm{1,2},\mathrm{3,4}\}$ denotes the response), then the MRP estimate for the intent to vaccinate within each of the UK’s 174 NUTS-3 regions is,

$${\Phi }_{jk} = \mathop \sum \limits_{s} \frac{{N_{s} \theta_{sjk} }}{N}.$$

In the main text, this quantity ${\Phi }_{jk}$ is computed for $k=4$ (“yes, [I] definitely [would accept a COVID-19 vaccine]”). Estimates are computed for those who are “unsure” (“unsure, but leaning towards yes” and “unsure, but leaning towards no” have been combined) and are shown in Fig. S1 in the supplementary materials. A similar equation to that above can be used to obtain the national-level MRP estimates in Fig. 4, but replacing population counts within each region with national population counts.

### Model: Implementation and output

The multilevel regression model detailed above is implemented using JAGS version 4.3.0 (implemented via rjags^52^) and R version 4.0.3. 25,000 posterior samples (excluding the first 5000 for model burn-in) was sufficient for successful convergence and all posterior draws were well-mixed. The posterior draws for the fixed effects are shown in Figure S4 and all look visibly well-mixed and all except “other work status” (*p* = 0.04) have Geweke *p*-values above 0.05. There are too many posterior draws to plot for all random-effects, but we show posterior draws for the first UK NUTS-3 region alphabetically (Hartlepool and Stockton-on-Tees) in Figure S5 with a histogram of Geweke *p*-values for all model parameters (fixed effects, random effects, and variance components) to demonstrate universally good mixing and convergence in Figure S3. In the computation of the Geweke statistic, the first 10% and final 50% of the posterior samples used for computation are used. Convergence of variance parameters is shown in Figure S6. A slightly larger fraction of Geweke *p*-values fall below 0.05 than is expected by chance (0.082 compared to 0.05 by chance). Manual inspection of these chains revealed no cause for concern: chains showed no ill-mixing or convergence issues.

### Eithics approval

Approval for this study was obtained via the Imperial College Research Ethics Committee on 24 July 2020 with reference 20IC6133 and European Union GDPR guidelines were followed throughout.

## Supplementary Information

Supplementary Information.

Supplementary Information.

## Data Availability

All survey data used in this study is available at https://osf.io/54n68/. UK Census Microdata is available at https://www.ons.gov.uk/census/2011census/2011censusdata/censusmicrodata.
